# The Role of DNA in the Extracellular Environment: A Focus on NETs, RETs and Biofilms

**DOI:** 10.3389/fpls.2020.589837

**Published:** 2020-12-17

**Authors:** Francesco Monticolo, Emanuela Palomba, Pasquale Termolino, Pasquale Chiaiese, Elisabetta de Alteriis, Stefano Mazzoleni, Maria Luisa Chiusano

**Affiliations:** ^1^Department of Agricultural Sciences, Università degli Studi di Napoli Federico II, Portici, Italy; ^2^Department of Research Infrastructures for Marine Biological Resources, Stazione Zoologica “Anton Dohrn”, Naples, Italy; ^3^Institute of Biosciences and Bioresources, National Research Council, Portici, Italy; ^4^Department of Biology, University of Naples Federico II, Naples, Italy

**Keywords:** self-DNA, exDNA, extracellular matrix, self-DNA inhibitory effect, exDNA as a DAMP

## Abstract

The capacity to actively release genetic material into the extracellular environment has been reported for bacteria, archaea, fungi, and in general, for microbial communities, but it is also described in the context of multicellular organisms, animals and plants. This material is often present in matrices that locate outside the cells. Extracellular matrices have important roles in defense response and disease in microbes, animal and plants cells, appearing as barrier against pathogen invasion or for their recognition. Specifically, neutrophils extracellular traps (NETs) in animals and root extracellular traps (RETs) in plants, are recognized to be important players in immunity. A growing amount of evidence revealed that the extracellular DNA, in these contexts, plays an active role in the defense action. Moreover, the protective role of extracellular DNA against antimicrobials and mechanical stress also appears to be confirmed in bacterial biofilms. In parallel, recent efforts highlighted different roles of self (homologous) and non-self (heterologous) extracellular DNA, paving the way to discussions on its role as a “Damage-associated molecular pattern” (DAMP). We here provide an evolutionary overview on extracellular DNA in extracellular matrices like RETs, NETs, and microbial biofilms, discussing on its roles and inferring on possible novel functionalities.

## Introduction

The presence of extracellular materials, organized as extracellular matrix (ECM), glycocalyx, or mucus layers, has been described in both vertebrates (Huxley-Jones et al., 2007; Möckl, 2020) and invertebrates (Har-el and Tanzer, 1993; Schröder and Bosch, 2016) as well as in plants (Driouich et al., 2013) and microorganisms (Flemming and Wingender, 2010). Despite the specific components may vary between clades or species, also in dependence of the specific cell types, the extracellular organization mostly shares gel-like structures mainly composed of glycoproteins, proteoglycans, and glycolipids (Theocharis et al., 2016).

The extracellular structures may fulfill relevant roles in terms of structure and functional organization, contributing to fundamental processes like cell adhesion, migration, proliferation, differentiation, and apoptosis. They can act as protective barriers in preventing pathogen invasion, or represent advantageous habitats to facilitate symbiotic interactions, for example favoring adhesion of microbial communities (Yue, 2014; Schröder and Bosch, 2016; Möckl, 2020).

In vertebrates, among interesting examples of specialized ECM production, there are those from cell types like fibroblasts, contributing to the organization of the connective tissue, chondrocytes, producing cartilage, and osteoblasts, producing the bone matrix (Alberts et al., 2002). Noticeable, also neutrophils, which are terminally differentiated killer cells in vertebrates (Dale et al., 2008) and invertebrates (Jenne et al., 2018), essential for both the innate and acquired immune systems (Mantovani et al., 2011; Rosales et al., 2017), are known to form specialized extracellular organization in terms of web-like structures, that are called neutrophil extracellular traps (NETs), and appear to have a relevant protective role against pathogens.

Similar structure organizations have been also described in plants. For example, the root extracellular traps (RETs), by analogy with the NETs, are identified as high molecular weight compounds surrounding the plant root cap. They are mostly composed by carbohydrates, and are produced by the root border cells, playing a crucial role in plant defense (Hawes et al., 2011, 2016; Driouich et al., 2013).

The presence of extracellular structures has been extensively described also in the microbial world, in microalgae, fungi, bacteria, and archaea. These structures are generally associated with biofilm formations. Indeed, biofilms are defined as an agglomerate of microorganisms hold in a self-produced ECM. Biofilm formation allows single cell microorganisms to acquire a temporary multicellular lifestyle, facilitating survival in specific conditions, or under specific environmental changes (e.g., levels of oxygen and/or carbon; Monds and O’Toole, 2009; Kostakioti et al., 2013), with possible roles in the increase of microbial fitness and protection, as examples, from predation, desiccation, starvation, and exposure to antimicrobials (O’Toole, 2003).

We here propose an overview of the current knowledge on the role of extracellular DNA (exDNA) in extracellular matrices like NETs, RETs, and biofilms, highlighting specificity and conserved traits among different clades. The role of exDNA in these matrices is also discussed in relationship with the evidence of the inhibitory role of conspecific exDNA on cell growth (Mazzoleni et al., 2015a, b), thus suggesting possible additional functions for DNA in extracellular matrices.

### Extracellular DNA

One of the current definitions of exDNA is “… located outside the cell and originating from intracellular DNA by active or passive extrusion mechanisms or by cell lysis” (Ceccherini et al., 2009).

Extracellular DNA is abundant in many habitats, including soil, sediments, oceans, and freshwater as well as the intercellular milieu of metazoan (Nagler et al., 2018). In all these contexts, the exDNA results from either cell lysis or active release, and can be found in both the double and single stranded, as well as more or less fragmented forms (Levy-Booth et al., 2007; Ceccherini et al., 2009; Thierry et al., 2016; Nagler et al., 2018). The fate of exDNA may include biotic degradation (mainly due to ubiquitous extracellular and cell-associated DNases) and abiotic (physical and chemical) decay, as well as environmental long-term preservation and possible incorporation by microbial cells or other living beings via horizontal gene transfer (HGT; Levy-Booth et al., 2007; Nielsen et al., 2007; Torti et al., 2015). Interestingly, the released DNA may also become part of extracellular structures, such as NETs (Brinkmann et al., 2004), RETs (Driouich et al., 2013), and biofilms (Whitchurch et al., 2002).

The presence of exDNA in the *Pseudomonas aeruginosa* biofilm was demonstrated in 2002, by Whitchurch et al. (2002), whose experiments highlighted the structural role of DNA in the establishment and development of the bacterial biofilm. In 2004 the presence of both chromatin and DNA (Brinkmann et al., 2004) was confirmed also in the context of NETs. Finally, in plant slime surrounding roots, later called RET (Driouich et al., 2013), the presence of the histone H4 was revealed in 2007 (Wen et al., 2007b) and, 2 years later, the co-presence of DNA macromolecules was demonstrated too. Thus, DNA resulted to be an essential structural component of the ECM in plants (Wen et al., 2009a).

### ExDNA as a DAMP

DNA in extracellular environment has often been discussed for its contribution to “Damage-associated molecular patterns” (DAMPs), also known as “danger-associated molecular patterns,” i.e., as a molecule of endogenous origin that, if present in the inappropriate compartment, is recognized as a self-damage and can initiate and perpetuate a non-infectious inflammatory response (Seong and Matzinger, 2004; Roh and Sohn, 2018). Indeed, after being released from damaged or dying cells, DAMPs may activate the innate immune system by interacting with pattern recognition receptors (PRRs; Roh and Sohn, 2018).

In animals, self-DNA of nuclear or mitochondrial origin is frequently reported to act as a DAMP and to determine various types of diseases. For instance, extracellular self-DNA is associated to several diseases and/or to their severity, like in cancers (Hawes et al., 2015), hypertension (McCarthy et al., 2015), and Parkinson and Alzheimer diseases (Lowes et al., 2020). Self-DNA is also considered to be involved in autoimmune diseases such as in rheumatoid arthritis (Rykova et al., 2017), in systemic lupus erythematosus (Barrat et al., 2005), and in other autoimmune diseases (Vakrakou et al., 2018).

In plants, it is well established that non-self-DNA (heterologous, i.e., DNA from phylogenetically unrelated species, or, more in general, distant in sequence similarity terms) of bacterial origin, triggers immunological responses with the formation of reactive oxygen species (ROS) and callose deposition (Yakushiji et al., 2009). Moreover, recent studies clearly indicated that the self-exDNA has specific effects in plants. In 2015, Mazzoleni et al. (2015a) reported evidence that fragmented exDNA accumulating in litter during the decomposition process, produces a concentration dependent, species-specific inhibitory effect, reducing root growth and seed germination of conspecifics. They highlighted for the first time that the exposure to fragmented self-DNA inhibits root growth in plants, while non-self-DNA does not trigger similar effects (Mazzoleni et al., 2015a). The authors suggested that the inhibitory effect could depend on the sequence similarity of the plant DNA with the one representing the fragmented molecules, since the toxic effect was also evident, although to a lower extent, when exposing plants to decomposing litters of phylogenetically similar plants. These studies paved the way to further investigations on possible novel roles of exDNA in ecology, plant physiology, and in translational research. Indeed, in 2016, Barbero and colleagues demonstrated that the treatment with fragmented self-DNA triggers specific early immune signaling responses in plants. Indeed, the authors showed that fragments of self-DNA, and not of non-self-DNA, induced intracellular calcium signaling and plasma membrane depolarization in *Phaseolus lunatus* and *Zea mays* (Barbero et al., 2016). Furthermore, in 2018, Duran-Flores and Heil demonstrated that in *Phaseolus vulgaris*, the exposure to self-DNA inhibits seed germination and triggers H_2_O_2_ production, mitogen-activated protein kinase (MAPK) activation, extrafloral nectar release (typical of the defensive response to herbivores) in combination with a decreased susceptibility to infection by the bacterium *Pseudomonas syringae* (Duran-Flores and Heil, 2018). In 2018, Vega-Muñoz et al. (2018) suggested that the response to self- and non-self-DNA could depend on the degree of self damage detected by the plant, confirming that, in line with previous findings (Mazzoleni et al., 2015a, b; Barbero et al., 2016; Duran-Flores and Heil, 2018), this could depend on the concentration of either self-DNA or non-self-DNA and on the phylogenetically distance of non-self-DNA. Vega-Muñoz et al. (2018) also suggested that the exDNA methylation patterns could explain the mechanism for self-DNA recognition in plants.

However, despite these evidences, the mechanisms behind the differential response of plants to self- and non-self-DNA remains still unclear, to our knowledge.

Bacteria can detect foreign DNA and thus activate specific responses, as it will be discussed later, however, the role of exDNA as a DAMP has never been proposed in bacteria.

The discovery of Mazzoleni et al. (2015a) was also extended by the same authors to different organisms other than plants, including microbes, fungi, protozoa, and insects (Mazzoleni et al., 2015b). Noticeable, these studies demonstrated that the toxic effect due to exposure to self-DNA (conspecific or similar/homologous) in plants is a general phenomenon, that appear to be a typical response in all species in all kingdoms, paving the way to further studies that could address the role and the molecular mechanisms involved in self-exDNA sensing.

### ExDNA Sensing

The exDNA has been demonstrated to be sensed in animals by receptors located in various cellular compartments, such as the nucleus (Brázda et al., 2012; Wang et al., 2019), the cytoplasm (Hornung et al., 2009; Herzner et al., 2015; Szczesny et al., 2018), and the endosomes (Hemmi et al., 2000).

The distinction between self and non-self DNA is also a relevant aspect to carefully consider when describing crucial processes related to the detection of exogenous DNA components. For example, the specific recognition of unmethylated CpG-rich DNA in the endosomal compartment is ascribed to the TLR9 receptor (Barton et al., 2006). CpG methylation patterns are typically underrepresented in bacteria genomes and this allows their fragments to be detected by the host. Interestingly, the underrepresentation of CpG methylation is also typical in the mitochondrial DNA (mtDNA), and its erroneous recognition as a foreign molecule can give rise to inflammatory and autoimmune pathological responses in animals (Barrat et al., 2005; Zhang et al., 2010). In the cytoplasm, the receptor cGAS is able to bind DNA in a sequence-independent manner, and preferentially binding long dsDNA or short dsDNA with unpaired open ends containing guanosines (Y-form DNA), that are primarily found in viral genomes (Herzner et al., 2015), thus favoring the recognition of non-self DNA sequences when present.

In plants, no specific DNA receptor has been reported yet. Nevertheless, it is suggested that the exposure to both self- and non-self-DNA induces an immunological response (Duran-Flores and Heil, 2015; Heil and Vega-Muñoz, 2019). It has been suggested that the recognition of exDNA in plants could involve a membrane-bound exDNA receptor that, upon recognition, triggers a downstream signaling cascade, or a membrane-bound exDNA transporter or channel, and/or a vesicle-mediated internalization that, after the exDNA internalization, could favor the detection via an intracellular sensor (Bhat and Ryu, 2016). The sensing of exDNA molecules has also been ascribed to mechanisms similar to the “well-known processes of interference, based on sequence-specific recognition of small-sized nucleotide molecules” (Mazzoleni et al., 2015a), that could justify the specific inhibitory roles of extracellular self-DNA. Some plant membrane proteins are considered good candidates as exDNA receptors (Bhat and Ryu, 2016). During infection, plants release defense proteins [pathogen-related (PR) proteins] in the extracellular environment. Certain PR proteins, such as Vpr10.1 and GaPR10, show RNase and DNase activities *in vitro*, and have a putative adenosine triphosphate (ATP)-binding domain (Xu et al., 2014). For their activities, they are considered potential candidates intercepting exDNA and/or extracellular RNA outside the cell. Interestingly, the treatment of Arabidopsis with dsRNA leads to impairment in a pathogen associated molecular pattern (PAMP)-triggered immunity response (Niehl et al., 2016). The membrane-bound SERK1 was suggested to be the potential dsRNA receptor in this process. Moreover, a transcriptome analysis of plants treated with bacterial RNA revealed over expression of ribonuclease (RNS)-1 (Lee et al., 2016). RNS1 is a member of the T(2) family of RNS proteins that are typically expressed in response to wounding in *Arabidopsis thaliana*, and this process is independent from the activation of the jasmonic acid and abscisic acid pathways, which are typical elicited by wound response. In addition, plant PRRs recognize danger signals both from self-damage and/or non-self-organisms (Medzhitov and Janeway, 2000; Seong and Matzinger, 2004). Among PRRs, surface-localized proteins, characterized by leucine-rich repeats (LLRs) motifs, have been proposed as putative exDNA receptors (Heil and Land, 2014). Nevertheless, all these evidences need additional investigations to further elucidate candidates exDNA receptors, their structure, and roles (Gallucci and Maffei, 2017).

In bacteria, the perception and recognition of exogenous DNA also occurs. In order to recognize foreign DNA, such as the viral genomes, bacteria may recognize differential patterns in DNA structure. Usually unmethylated or differently methylated DNA of exogenous DNA are recognized through the DNA restriction-modification system (Wilson and Murray, 1991) and/or by the CRISPR–Cas systems (Dupuis et al., 2013; Shah et al., 2013; van der Oost et al., 2014). *Cis* elements as the *Chi* sequences may be recognized by the RecBCD recombination system, and may characterize the bacterial DNA because of their higher frequency and their absence in phages (Vasu and Nagaraja, 2013). In addition to the above-mentioned defense systems, bacteria can also keep track of invasive elements by specific transcriptional silencing of horizontally acquired genes or prophages recombined with their own genome through the recognition of different compositional patterns, such as the higher A-T contents in foreign molecules, and thus silencing them by the binding of repressor proteins (i.e., the heat-stable nucleoid-structuring protein (Navarre et al., 2007) or through the action of transcription termination factor (i.e., Rho protein; Cardinale et al., 2008). Interestingly, during the transformation process, the DNA uptake in most systems appears not to be sequence-specific. However, in some Gram-negative bacteria, such as *Haemophilus influenzae* (Sisco and Smith, 1979; Elkins et al., 1991) and *Neisseria species* (Danner et al., 1980; Fitzmaurice et al., 1984; Goodman and Scocca, 1988), the DNA uptake is more efficient if specific sequences called DUS (DNA uptake sequences), are present. As the genomes of these bacteria are enriched in their respective DUS (Smith et al., 1995; Parkhill et al., 2000; Tettelin et al., 2000), the uptake of self DNA is favored. Nevertheless, specific DUS receptors on the bacterial surface have not yet been identified (Chen and Dubnau, 2004).

DNA receptors have also been identified in bacteria. In gram-positive bacteria, such as *Bacillus subtilis* and *Streptococcus pneumoniae*, the DNA-binding protein ComEA is considered a DNA receptor (Inamine and Dubnau, 1995; Bergé et al., 2002). In gram-negative bacteria, such as in *Neisseria gonorrhoeae*, orthologs of the protein ComEA contain the DNA-binding domain (Chen and Gotschlich, 2001) and, presumably, may have the same role. Recently, the protein ComH has been identified in the gram negative bacteria *Helicobacter pylori* as a periplasmic DNA-binding protein, that interacts with the periplasmic domain of the inner membrane translocator ComEC to transfer the DNA into the cytoplasm (Damke et al., 2019).

## NETs

**FIGURE 1:**
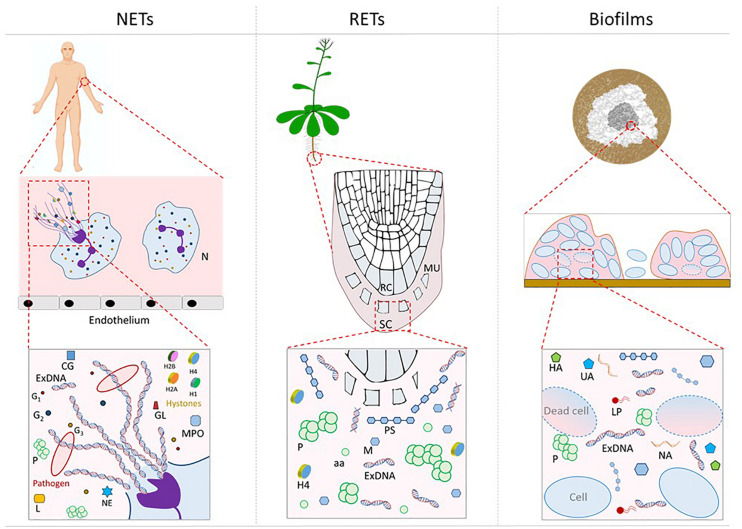
Schematic representation of NETs (animals), RETs (plants), and biofilms (microbial communities) structures. NETs: N, neutrophil; MPO, myeloperoxidase; NE, neutrophil elastase; Gn, neutrophil granules; H1, H2A, HAB, H4, histones; CG, cathepsin G; L, lactoferrin; GL, gelatinase; RETs: RC, root cap cells; MU, mucilage; SC, sloughed cells; aa, aminoacids; Biofilms: NA, nucleic acids; LP, lipids, UA, uronic acid; HA, humic acid; ExDNA, extracellular DNA; M, monosaccharides; PS, polysaccharides; and P, proteins.

Extracellular traps produced by eosinophils, mast cells, macrophages, and neutrophils are extracellular components in animals that have been demonstrated to contain DNA in their structure organization (Goldmann and Medina, 2012). Among these, NETs that are produced by neutrophils, are the most studied extracellular traps (Figure 1). Neutrophil cells, designed as heterophils in birds, reptiles, and some mammals, are the most abundant granulocytes (Montali, 1988), representing from 40%, up to 70%, of all white blood cells in humans. They are also present in higher invertebrates in the form of primordial neutrophils (Jenne et al., 2018), where they play active roles in the process of phagocytosis, but also share the ability to form clotting of haemolymph with platelets.

Mature neutrophils are released from bone marrow into the bloodstream (Mayadas et al., 2014), and represent the first line of defense against the invading microbes (de Bont et al., 2019). Indeed, they kill microbes by releasing proteases that favor their engulfment by macrophages, through phagocytosis, activating the immune system (Sofoluwe et al., 2019).

Neutrophils extracellular trapsosis is the process by which the NET formation occurs. This process was described for the first time in 2004 by Brinkmann et al. (2004). It consists in the expulsion of DNA, proteases, and antimicrobial peptides into the extracellular space (Sofoluwe et al., 2019). In particular, the induction of NETosis activates the nicotinamide adenine dinucleotide phosphate (NADPH) oxidase complex that produces superoxide anions. Superoxide anions are converted into hydrogen peroxide, which is a substrate of the myeloperoxidase (MPO) that induces the release of the neutrophil elastase (NE) from neutrophil granules. NE and MPO migrate to the nucleus, where they induce histone degradation decondensing chromatin structure. Moreover, NE degrades actin filaments inhibiting neutrophils movement (Fuchs et al., 2007; Papayannopoulos et al., 2010; Metzler et al., 2011, 2014; Palmer et al., 2012; de Bont et al., 2019). Therefore NETs composition consists in proteins from primary, secondary, and tertiary neutrophils granules, MPO, NE, DNA, and histones (H1, H2A, H2B, H3, and H4), in addition with cathepsin G, lactoferrin, gelatinase, as initially revealed by Brinkmann et al. (2004).

DNA in NETs can be composed either by nuclear or mitochondrial DNA. In 2009, Yousefi et al. (2009) showed that, in specific conditions, NETs could be formed from pure mtDNA, and this was not accompanied by NETosis since neutrophils remained viable. In 2014, McIlroy et al. (2014) demonstrated that NETs could be released also after injury and orthopedic trauma surgery.

NETs influence the humoral innate immunity by producing part of the complement factors cascade (de Bont et al., 2019), which consists in more than 30 proteins mostly produced by the liver. They are activated by a sequence of proteolytic cleavages ending with the formation of a pore on the pathogen cell membrane that, losing its integrity, determines pathogen death (Sarma and Ward, 2011; Janeway et al., 2017). Interestingly, complement activation by NET formation is strongly decreased by DNase I (de Bont et al., 2019). Furthermore, NETs can also act as a scaffold for clot formation, highlighting novel insights on the role of neutrophils and NETosis in coagulation-mediated diseases (de Bont et al., 2019).

Neutrophils extracellular traps are structures including active molecules with strong intermolecular bindings, preventing their diffusion into neighboring tissues. *In vivo*, NETs are degraded by DNases and removed by macrophages (Hakkim et al., 2010; Farrera and Fadeel, 2013; Jiménez-Alcázar et al., 2017; de Bont et al., 2019). These are essential steps that follow NETs formation and are required for NETs clearance. Remarkably, in 2017, Jiménez-Alcázar et al. (2017) demonstrated that DNase 1 and DNase 3 are essential for NET clearance, and that mice deficient in DNase 1 and DNase 3 die few days after neutrophil activation, because of blood vessels occlusion caused by persistent NET structures (Jiménez-Alcázar et al., 2017). Moreover, Savchenko et al. (2014) in their studies on the role of innate immune cells in the early response to myocardial ischemia/reperfusion injury, demonstrated that myocardial injury caused an increase in nucleosomes, neutrophil infiltration, and histone H3 at the site of injury. Treatment with DNase improved cardiac contractile function to a similar degree in both wild type and PAD4-/- deficient mice, which do not produce NETs. This suggested that DNA fragments contribute to cardiomyocyte dysfunction irrespective of NETs, possibly by acting as DAMPs (Savchenko et al., 2014; Shah et al., 2020). Confirming its positive role against NET-mediated pathologies, DNase 1 has been proposed as an enzyme able to attenuate them in mice (Németh et al., 2020). Consistent with the observation in mice, the persistence of NETs can have serious negative consequences in humans, leading to pathologies such as cardiovascular, lung and eye diseases, atherosclerosis, rheumatoid arthritis, thrombosis, diabetes, cancer, and severe COVID-19 (Demers et al., 2012; Arazna et al., 2013; Brinkmann, 2018; Daniel et al., 2019; Erpenbeck et al., 2019; Leppkes et al., 2020).

In 2020, Neumann et al. (2020) traced the evolutionary presence of NET like structures, organized by the extrusion of decondensed chromatin and additional intracellular material, in different phyla: Chordata, Arthropoda, Mollusca, Cnidaria, and the Plantae kingdom included. However, the functional role of their presence is still questionable, as also commented by the authors themselves: “can organisms from other life kingdoms use a similar mechanism as defense strategies against their foes?” (citation by Neumann et al., 2020).

Notwithstanding the great interest and relevant roles of NETs, their release mechanisms are not fully understood and some aspects of the process still remain unclear (Manda-Handzlik et al., 2019). In addition, the structure and possible functional roles of exDNA in NETs organization is still matter of investigation.

## RETs

Plant roots provide water and nutrients to the whole plant body. They show a peculiar organization that is also determined by specific assemblages of extracellular materials, mainly represented by root mucilages. In the external part of the root apex, adjacent to the apical meristem, the plant root cap represents a dynamic and multifunctional tissue. This tissue is extremely resistant to both biotic and abiotic stimuli, in contrast with the internal, highly proliferating, tissue that represents the root elongation zone. The peculiar resistance of the root cap tissue depends on the presence of root border cells at the cap periphery. These cells, in most plant species, separate from the cap as a metabolically active population of cells, that is released into the rhizosphere as free cells or in clump (Brigham et al., 1998; Gunawardena and Hawes, 2002; Wen et al., 2007b; Hawes et al., 2016). Originally, border cells were defined as those cells that are released into suspension by a brief immersion of the root tip into water (Brigham et al., 1995). Proteomic and gene expression profiling studies revealed that these cells are different from their progenitors in the root cap, although they share similarities across diverse plant species (Brigham et al., 1995; Wen et al., 2007b, 2009b). Root border cells were previously referred as “sloughed root cap cells,” since they were thought to be a product of tissue disintegration. Subsequently, they were termed “border cells” to emphasize that they are viable after the detaching from the root cap and that they are a specialized tissue, morphologically and physiologically different from the root cap cells (Hawes et al., 2016).

It has been shown that root cap cells and border cells are able to secrete the root mucilage, the high molecular weight sticky matrix that surrounds the plant root cap, through an active continuous process, that piles up materials outside the root (Figure 1). The number of border cells and their secretion into the rhizosphere can vary according to many factors [water availability, soil type, physical abrasion, day length, root age, growth rate, the amount of carbon dioxide, of aluminum, of boron, plant pathogens, the altered expression of genes controlling cell cycle or cell wall solubilization at the cap periphery (Hawes et al., 2012)]. Moreover, root mucilage formation also contributes to the whole root network asset, starting from an initial structure surrounding the root cap (Driouich et al., 2019), and in relationships with growth conditions, that determine the root mucilage secretion (Hawes et al., 2012).

The root mucilage is mostly composed by both mono and polysaccharides (mainly galactose, glucose, arabinose, fucose, and xylose), proteins (e.g., proteases, peroxidases, plant defense-related proteins, such as defensins, well-known to be also relevant components of the plant cell wall and of the apoplast) and amino acids. Interestingly, the root mucilage was also revealed to be formed by known intracellular markers, such as histone H4 (Wen et al., 2007b; Weiller et al., 2017). Together with the histone H4, the presence of DNA in the root mucilage was also reported (Wen et al., 2009a). Other molecules could be also part of root mucilage (Vincent et al., 2020).

Plant exDNA in RETs was initially thought to be derived by leakage from dead cells (Levy-Booth et al., 2007). However, currently, there is no observation demonstrating that exDNA in RETs is released by lytic processes (Driouich et al., 2019). Indeed, it has been recently demonstrated that newly synthesized DNA is actively exported into the ECM by vital root cap cells, even if the leakage of nuclear content from dead cells cannot be excluded (Wen et al., 2009a). Once released, the exDNA forms distinctive structures, similar to those produced by neutrophils (Patel et al., 2010; Pilsczek et al., 2010). In addition, initial analyses revealed that the exDNA in RETs is mainly represented by nuclear DNA enriched in repetitive sequences (Hawes et al., 2012) and, moreover, to date there is not yet evidence of presence of mtDNA sequences in these structures (Driouich et al., 2019).

In 1942, Rogers and his colleagues advanced the hypothesis that the border cells producing the root mucilage may represent an “extra-root” digestive system (Rogers et al., 1942), that functions as an exoenzyme system releasing substances, like phosphatases, into the rhizosphere (Driouich et al., 2019). This putative function could resemble the well-known extracellular digestive activity before substrate absorption in fungi, during organic matter decomposition processes (Jennings, 1995; Cole, 1996). Many other roles were associated with the root mucilage, such as: lubricant protecting the root tips while growing into the soil (Greenland, 1979); carrier of gravitropic signals from the root cap to the root tip (Moore et al., 1990); protection of roots from the toxicity of ions such as copper, cadmium, boron, lead, mercury, iron, arsenic, aluminum (Mench et al., 1987; Hawes et al., 2016), or as carbon source for soil microbes (Knee et al., 2001).

The root cap secretion represents a primary site in the root that is colonized by microbial symbionts and pathogens that are present in the rhizosphere. On one hand, it is well known that the rhizosphere sheet surrounding the fine roots is a complex ecosystem, representing the habitat of specific microbial communities interacting with the plant, including bacteria and mycorrhizal organisms in both symbiotic and mutualistic relationships with the root (Lambers et al., 2009; McNear Jr, 2013). On the other hand, similarly to NETs in animals, diverse plant pathogens interact with border cells, which appear to act as a trap against microorganisms, forming aggregates and inhibiting pathogen growth. It has been proposed that the root slime works by “trapping” pathogens to protect the root tip meristem, whose function is critical to root development and plant survival and with a structure that does not show specific resistance to biotic or abiotic stress (Whipps, 2001; Raaijmakers et al., 2009).

Interestingly, the extracellular trapping phenomenon is host-microbe specific, with no aggregation or growth inhibition of non-pathogenic organisms (Jaroszuk-Ściseł et al., 2009). The chemotaxis and the binding of host–specific microbes (bacteria, as well as nematodes, zoospores, and fungi), along the plant cell wall and on the structures of the mucilage secreted by border cells, are always followed by quiescence of the pathogen population (Wen et al., 2017).

All the constituents of the RETs play an important role in the host defense against pathogens. For example, the importance of the involved proteins was documented by the fact that, when the roots are treated with proteases at the time of inoculation with spores of a pathogenic fungus, the normal resistance to root tip infection is abolished (Wen et al., 2007b). Treatment with proteases also results in the disintegration of the surrounding mucilage layer with the subsequent release of bacteria within the layer (Wen et al., 2007a). This evidence suggests that proteins may play a role in the structural integrity of the matrix (Matsuyama et al., 1999), even though they comprise only a small fraction of the matrix composition, which is mainly composed by carbohydrates (Bacic et al., 1986; Moody et al., 1988; Chaboud and Rougier, 1990; Hawes et al., 2012).

Furthermore, the degradation of exDNA results in loss of root tip resistance to infection. When DNase 1 is added at the time of pathogen inoculation, 100% of root tips becomes necrotic within 48–72 h (Hawes et al., 2011). Moreover, the inactivation of extracellular DNases in the plant pathogen *Ralstonia solanacearum* reduces the virulence, showing that the infection is related to the pathogen capability of dissolving the structural organization of the extracellular trap, thus reducing its protection function (Tran et al., 2016; Wen et al., 2017).

Despite the reported evidence, mechanisms of RETs formation and root mucilage depositions, as well as those promoting the DNA release by the border cells into the extracellular space, still need to be completely elucidated.

## BIOFILMs

It is known that the microbial world can appear organized in specific structures composed of sessile cells encased by an ECM, which are called biofilms (Figure 1).

The first observation of microbial biofilms was made by Antonie van Leeuwenhoek, in 1684, when he found aggregates of different microbes colonizing his own teeth and tongue (Dobell, 1932). Later, Pasteur observed that aggregates of microbes allowed the fermentation of wine into vinegar (Pasteur, 1864). In the following years, researchers lost interest in biofilms until 1985 (Høiby, 2017), when it was demonstrated the increase in the antimicrobial resistance of biofilm-enclosed bacteria compared to the planktonic counterparts (Nickel et al., 1985). Since then, the interest in biofilm research enormously increased, also because the biofilm life-style was recognized to be the most common mode of growth and survival of microbial species in the environment, with huge implications in ecology, industry, biotechnology, and clinics.

The biofilm formation is a reversible process in which cells can return to planktonic life-style if perturbed by hydrodynamic and repulsive forces, or as a consequence of nutrient depletion (Donlan, 2002). Biofilm development is determined by both intrinsic and environmental factors and consists of different stages. It starts from single cells on a surface showing a stochastic distribution (Kostakioti et al., 2013; Armbruster and Parsek, 2018). On the surface, cells encounter attractive or repelling forces depending on environmental conditions, such as nutrient availability, ionic strength, pH, and temperature. These factors affect the velocity and the direction toward or away from the contact surface (Donlan, 2002). Once microorganisms adhere to the surface, the attachment becomes stable, cells start multiplication and secretion of the ECM, that is also named extracellular polymeric substance (EPS; Flemming and Wingender, 2010). This process leads to the formation of micro-colonies (Costerton et al., 1999). The biofilm architecture can favor different processes such as the exchange of nutrients, the distribution of metabolic products, and of signaling molecules (Jamal et al., 2018). Microbial cells communicate with each other through auto-inducer signals during biofilm maturation, which affect the microbial cell density (Davies et al., 1998; Costerton et al., 1999; Federle and Bassler, 2003). During the maturation, the EPS becomes essential for the biofilm three-dimensional structure organization and for the survival of the micro-colonies. In fact, interstitial channels are embedded in the EPS acting as a circulatory system that favors the distribution of nutrients and the removal of waste products (Jamal et al., 2018). The final stage in the biofilm life cycle includes the production and release of dispersal cells which switch from sessile into motile forms. They leave the original microcolonies and can colonize new surfaces to initiate the surface-association phase of the biofilm formation (McDougald et al., 2012).

The EPS of a biofilm may differ depending on the species, but it is generally composed by several molecules such as carbohydrates, lipids, proteins, and nucleic acids, including DNA (Zhang et al., 1998; Flemming and Wingender, 2010; Kassinger and van Hoek, 2020) as well as by pili, flagella, humic, and uronic acids, which are all considered essential components of the biofilm organization (Nielsen et al., 1996). Extracellular carbohydrates in the biofilm matrix can trap micronutrients, enhance the attachment to the surface and biofilm formation (Harrison et al., 2007; Kassinger and van Hoek, 2020). Extracellular vesicles have also been found in the biofilm matrix of different microbial species, contributing to its lipidic and protein content (Schooling et al., 2009; Zarnowski et al., 2018; Kassinger and van Hoek, 2020). Nevertheless, the role of all these components in the biofilm organization is still under investigation.

Beyond its structural and functional role during cell adhesion and biofilm development, the extracellular DNA has never been discussed for its role as a DAMP in bacteria, also in the context of biofilm formation.

## Role of DNA in the NETs, RETs, and BIOFILMs

The presence of exDNA in NET, RET, and biofilm drives the attention on its structure and functional roles in each of the specific contexts (Table 1).

**TABLE 1:** Main roles of extracellular DNA in NETs, RETs, and Biofilms and associated bibliographic references confirming (Confirmed), hypothesizing (Hypothesis) the specific role or not available (n/a).

	Extracellular structure		
ExDNA role	NET	RET	Biofilm
Structure	Confirmed^1,2^	Confirmed^3–6^	Confirmed^7–24^
Defense	Confirmed^1,2,25–27^	Confirmed^3,28–31^	Confirmed^32,33^
Pathogen trap	Confirmed^1,2,25,26^	Confirmed^28–31^	n/a
Autotoxicity	Confirmed^2,27,34–43^	Hypothesis^44–48^	Hypothesis^45^
Source of genetic information	n/a	n/a	Confirmed^49–51^
Source of inorganic phosphate	n/a	Confirmed^52^	n/a

*Numbers are associated to the corresponding bibliographic references as follows: ^1^Brinkmann et al. (2004), ^2^Yousefi et al. (2009), ^3^Wen et al. (2009a), ^4^Hawes et al. (2012), ^5^Patel et al. (2010), ^6^Pilsczek et al. (2010), ^7^Kassinger and van Hoek (2020), ^8^Zhang et al. (1998), ^9^Ascher et al. (2009), ^10^Flemming and Wingender (2010), ^11^Flemming et al. (2016), ^12^Decho and Gutierrez (2017), ^13^Abada and Segev (2018), ^14^Mann et al. (2009), ^15^Whitchurch et al. (2002), ^16^Harmsen et al. (2010), ^17^Gödeke et al. (2011), ^18^Nguyen and Burrows (2014), ^19^Das et al. (2010), ^20^Seviour et al. (2019), ^21^Gloag et al. (2013), ^22^Peterson et al. (2013), ^23^Payne and Boles (2016), ^24^Gallo et al. (2015), ^25^Halverson et al. (2015), ^26^Baums and von Köckritz-Blickwede (2015), ^27^Tsourouktsoglou et al. (2020), ^28^Tran et al. (2016), ^29^Driouich et al. (2019), ^30^Plancot et al. (2013), ^31^Hawes et al. (2011), ^32^Chiang et al. (2013), ^33^Mulcahy et al. (2008), ^34^Savchenko et al. (2014), ^35^Shah et al. (2020), ^36^Krysko et al. (2011), ^37^Demers et al. (2012), ^38^Brinkmann (2018), ^39^Daniel et al. (2019), ^40^Erpenbeck et al. (2019), ^41^Leppkes et al. (2020), ^42^Arazna et al. (2013), ^43^Wang et al. (2015), ^44^Mazzoleni et al. (2015a), ^45^Mazzoleni et al. (2015b), ^46^Barbero et al. (2016), ^47^Duran-Flores and Heil (2018), ^48^Vega-Muñoz et al. (2018), ^49^Lorenz and Wackernagel (1994), ^50^Merod and Wuertz (2014), ^51^Orwin et al. (2018), and ^52^Paungfoo-Lonhienne et al. (2010).*

### DNA in NETs

In NETs, the DNA has been shown to provide a major contribution to the antimicrobial activity. Indeed, it possesses the ability to sequester surface bound cations, disrupt membrane integrity, and lyse bacterial cells (Halverson et al., 2015). The DNA antimicrobial property is determined by its direct contact with the bacterial membrane and by the phosphodiester backbone that is required for the cation chelation (Baums and von Köckritz-Blickwede, 2015). In fact, it has been demonstrated that treatment of NETs with an excess of cations or phosphatase enzyme, and exogenous or secreted microbial DNAses, protects pathogens from the NET antibacterial action (Baums and von Köckritz-Blickwede, 2015; Halverson et al., 2015). Furthermore, Halverson et al. (2015) demonstrated that the DNA in NETs induces the upregulation of protective surface modifications in bacteria (Halverson et al., 2015). In fact, bacteria co-incubated with NETs, upregulates the expression of the *arn* operone and of spermidine synthesis genes (Baums and von Köckritz-Blickwede, 2015). These two factors stabilize the bacterial envelope and mediate resistance to antimicrobial peptides (Johnson et al., 2012; Gutu et al., 2015). In 2020, Tsourouktsoglou et al. (2020) showed that histones and DNA work together in triggering inflammation, when histone-induced cytotoxicity is not reached. Indeed, at low concentrations, nucleosomes can induce cytokines, and the inflammatory response, whereas at high concentrations they kill the cells (Tsourouktsoglou et al., 2020). Cooperative effects due to histones and DNA are essential for the production of cytokines without killing cells. In fact, histones bind and activate TLR4, whereas DNA recruits TLR4 into endosomes containing histones (Tsourouktsoglou et al., 2020).

Of note, as mentioned above, NETs were demonstrated to be formed from pure mtDNA which can have a potent proinflammatory effect, acting as a DAMP, and directly modulating an inflammatory response (Krysko et al., 2011). This is due to the different methylation pattern of mtDNA when compared to nuclear DNA (Patil et al., 2019), making it detectable as a foreign molecule (bacterial or viral like), rather than a “self” DNA molecule (Yousefi et al., 2019), thus activating an immune response by stimulating the PRRs STING (Lood et al., 2016). Furthermore, in 2015, Wang et al. (2015) demonstrated higher levels of mtDNA in NETs of systemic lupus erythematosus patients when compared with controls, suggesting a possible role of mtDNA in autoimmune diseases (Wang et al., 2015).

### DNA in RETs

Similarly to NETs, both the histones H4 and the exDNA in RETs are suggested to have an antimicrobial activity (Tran et al., 2016; Driouich et al., 2019). The former, like the cationic antimicrobial peptides, may bind and disrupt microbial cell membranes. The DNA in RETs is discussed to have a structural role like a scaffold allowing the adhesion of anti-microbial components, being also considered as a trap for pathogens (preventing their spread throughout the organism). In addition, it exerts a direct bactericidal function (Halverson et al., 2015), putatively with the same action discussed for NETs. Evidence suggested that, in RETs, the DNA might be an integral component of plant defense, playing a relevant role in the innate immunity response to pathogen invasion. For instance, it is reported that the DNA is released in the extracellular environment with other molecules, such as callose, ROS, and cell wall extensins, in response to pathogen molecules (Plancot et al., 2013). Moreover, the production of extracellular DNases contributes to pathogens virulence (Hawes et al., 2011). The plant pathogen *R. solanacearum* produces two extracellular DNases that are able to degrade the DNA in pea root mucilage, allowing the pathogen to overcome the border cell trap. Conversely, *R. solanacearum* mutants, lacking both nucleases, remain immobilized in the root matrix, thus showing a reduced virulence (Steichen et al., 2011; Tran et al., 2016). Worthy to note, although the role of self exDNA as a DAMP has been discussed in plants (Barbero et al., 2016; Duran-Flores and Heil, 2018; Vega-Muñoz et al., 2018), as also here reviewed, there is no clear evidence that the exDNA released by root cap cells in the extracellular space and organized in RETs could act as a DAMP too, thus triggering an immunological response in plants.

Despite the evidence listed above, the role of exDNA in RETs remains to be further elucidated. Its release by viable border cells suggests an active role in plant root defense against pathogens in the rhizosphere. However, further analyses are still required to elucidate its possible functions and associated mechanisms.

### DNA in Biofilms

ExtracellularDNA is today accepted to be essential during biofilm formation and development (Ascher et al., 2009), as a component of the ECM in both terrestrial and marine biofilms (Flemming and Wingender, 2010; Flemming et al., 2016; Decho and Gutierrez, 2017; Abada and Segev, 2018), as well as in biofilms of clinically relevant microorganisms such as *Staphylococcus spp., Streptococcus spp., Candida spp., P. aeruginosa* (Mann et al., 2009). Interestingly, DNA is mostly represented by randomly fragmented genomic DNA (Steinberger and Holden, 2005; Wu and Xi, 2009; Kassinger and van Hoek, 2020).

The exDNA is released into the surrounding matrix not only by lysed cells (Sutherland, 2001), but also by an active release, sometimes mediated by membrane vesicles (Kadurugamuwa and Beveridge, 1995; Grande et al., 2015; Kassinger and van Hoek, 2020). In particular, the mechanism of DNA release differs among gram positive and gram negative bacteria. Gram positive bacteria are thought to release DNA in biofilms by autolysis or lytic processes, while the formation of vesicles and the release by the type 4 secretion system appear to be additional processes that are typically described in gram negative bacteria, depicting and active release of DNA in the extracellular space (Ibáñez de Aldecoa et al., 2017).

Multiple functions have been described for exDNA in biofilms (Das et al., 2013; Okshevsky and Meyer, 2015; Ibáñez de Aldecoa et al., 2017). The structural role of exDNA in biofilm formation (Whitchurch et al., 2002; Harmsen et al., 2010; Gödeke et al., 2011) has been demonstrated by the treatment with DNases, that generally lead to biofilm disruption and consequent cell dispersal (Whitchurch et al., 2002; Nguyen and Burrows, 2014). Furthermore, it has been demonstrated that exDNA forms complexes with the amyloid proteins secreted by different species, generating biofilms (Barnes et al., 2012; Schwartz et al., 2016). Apart from the structural role discussed above, other specific functions of exDNA in biofilms have been suggested. ExDNA may act as facilitator of the initial adhesion of cells to the surface (Das et al., 2010), in support of EPS gelification (Seviour et al., 2019), in the maintenance of specific cell orientations (Gloag et al., 2013), in the control of viscoelastic relaxation of the biofilm in mechanical stress conditions (Peterson et al., 2013) and in the induction of the morphological changes from yeast to hyphal growth, during *Candida albicans* biofilm development (Payne and Boles, 2016).

The presence of DNA in the matrix has been also related to biofilm antibiotic resistance (Chiang et al., 2013). For instance, a higher antimicrobial resistance has been detected in the presence of higher concentration of exDNA in biofilms (Mulcahy et al., 2008). Moreover, the chelator action of negatively charged exDNA phosphodiester backbone also plays a role against cationic antimicrobials (Mulcahy et al., 2008). Biofilms have also been shown to stimulate the host innate and the adaptive immune system, and this may cause the development or the progression of host autoimmune responses (Gallo et al., 2015). It has been suggested that these events could be favored by different putative causes. Indeed, the autoimmune response could be triggered or by the release of DAMPs, due to drastic damages of the host structures caused by the bacteria infection, and/or by components of their biofilm structure. As an example, bacteria biofilms may be composed by scaffolds of amyloid proteins that are highly resistant to degradation. For example, curli fibers are amyloids present in biofilms of enteric bacteria. It was shown that the complex between curli fibers and bacterial DNA in enteric biofilms has a higher inflammatory activity when compared with the effects of curli fibers or DNA alone (Gallo et al., 2015). It was demonstrated that curli fibers are detected by the TLR2/TLR1 heterocomplex on the membrane of immune cells. This triggers the internalization of the curli/DNA complex via endosome formation, and the activation of the receptor TLR9 by the bacterial DNA. Since amyloids are expressed also by human cells, their presence could trigger the production of autoantibodies, justifying the possible occurrence of autoimmune responses (Gallo et al., 2015). It has been suggested, however, that the autoimmunity response triggered by the exposure to bacteria biofilm may evolve in autoimmune disease when the individual is already predisposed by genetic factors. Beyond these effects, however, currently there is no further evidence, to our knowledge, that could suggest possible additional roles of biofilm components in causing autoimmune responses, rather than their capability to trigger the development of autoantibodies or trigger adverse reactions due to the detection of their DNA by host cell intracellular receptors.

The role of exDNA as a source of genetic information in the context of HGT within the biofilm has been addressed in several studies (Lorenz and Wackernagel, 1994; Merod and Wuertz, 2014; Orwin et al., 2018). Homologous recombination of foreign DNA into the host chromosome following transformation is believed to play a major role in bacteria evolution (Flemming et al., 2016). Biofilm offers ideal conditions for exchanges of genetic material because of the high cell density, increased genetic competence, and the presence of abundant exDNA.

From a clinical point of view, exDNA also turns out to represent a possible target for antibiotic agents acting against biofilm structural integrity, increasing the susceptibility of its constituents (Koo et al., 2017; Rocco et al., 2017; Ye et al., 2017).

## Discussion

Extracellular DNA, whether released in biofilm by bacteria, in RETs by root cap cells or in NETs by neutrophils or other cell types, has key structural, and functional roles that we here reviewed, as summarized in Figure 1 and Table 1. In addition, further intriguing roles of exDNA produced by an organism or by cells from the same species, have also been described in terms of extracellular self-DNA inhibitory effects.

In 2015, Mazzoleni demonstrated for the first time that the exposure to fragments of self-DNA, and not of non-self-DNA, inhibits root growth in plants in a concentration dependent manner (Mazzoleni et al., 2015a). Moreover, Mazzoleni and co-workers suggested that the phenomenon is dependent on the similarity of the “self” DNA fragments with the genome of the treated species, thus explaining the autotoxicity in litters of phylogenetic related species. Indeed, they demonstrated that in a specific plant species the treatment with different non-self-DNA fragments produces different effects: the closer the organisms phylogenetic distance, the higher the inhibitory effect of non-self-DNA on the treated plant (Mazzoleni et al., 2015a).

Following their experimental evidences, Mazzoleni and co-workers hypothesized that the inhibitory effect of self-DNA, because of its specificity and its occurrence also with DNA of related species, could be the result of a mechanism resembling the “well-known processes of interference based on sequence-specific recognition of small-sized nucleotide molecules” (Mazzoleni et al., 2015a).

Later, the same authors demonstrated that the inhibitory effect of self DNA, compared to non-self DNA, is a general phenomenon (Mazzoleni et al., 2015b) since the growth inhibition was also demonstrated for other species of different taxonomic groups (such as algae, bacteria, and fungi), reinforcing the hypothesis that the mixture of random self-DNA fragments could cause both an interference or exert inhibitory effect on the whole genome functionality (Mazzoleni et al., 2015a).

Duran-Flores and Heil (2015), considering the same topic, suggested two possible mechanisms: a self-specific membrane reception followed by a downstream signaling cascade activation or the direct uptake of fragmented DNA into cells with subsequent interference with essential biological processes. A further hypothesis was suggested to explain the dosage-dependent growth-inhibition by self-DNA as the phenotypic consequence of a costly immune response (Duran-Flores and Heil, 2015).

Cartenì et al. (2016) proposed that the first sensing of exogenous self-DNA could occur at the level of RET and, following its uptake, the cell functionality could be affected in different manners. For example, the self- DNA could interfere with gene expression following a sequence-specific recognition of homologous sequences recognition involving RNA/DNA interactions or the direct interaction with the genome structure through a mechanisms similar to the Small Fragment Homologous Replacement (Cartenì et al., 2016). They suggested that a mixture of random self-DNA fragments, once inside the plant cell, recognizes and anneals with the homologous DNA sequences in the plant genome. This could lead to the formation of structures that activate mechanisms of DNA repair allowing the integration of small DNA fragments into the genomic DNA thus affecting cellular activities (Cartenì et al., 2016).

Currently, however, the mechanisms underlying the specific recognition of either self and non-self-DNA and subsequent responses in plants are still poorly understood (Bhat and Ryu, 2016).

Beyond the structure and functional roles here reviewed for NETs, RETs, and biofilms, novel intriguing aspects arise when considering the specific inhibitory effects of extracellular self-DNA demonstrated by Mazzoleni et al. (2015a, b) and in subsequent efforts (Duran-Flores and Heil, 2018).

Concerning NETs, it is well known the role of the DNases responsible for their degradation that, while avoiding NET diffusion into neighboring tissues, favor their necessary clearance. Indeed, not removed NETs are able to form clots causing vascular occlusion and organ failure (Papayannopoulos et al., 2010; Arazna et al., 2013; Jiménez-Alcázar et al., 2017). Moreover, the evidence that in the absence of a complete NET removal, the complex of chromatin-DNA can be a primary target for autoantibodies leading to the development of autoimmune diseases (Barrat et al., 2005; Wang et al., 2015; Rykova et al., 2017; Vakrakou et al., 2018) or that DNA fragments contribute to cardiomyocyte dysfunction irrespective of NET formation (Savchenko et al., 2014; Shah et al., 2020), or that DNases can attenuate NET-mediated pathologies (Németh et al., 2020), indicates a potential negative effect of extracellular self-DNA present in NETs.

Indeed, the persistence of self-DNA in the context of NETs has been demonstrated to exert pro-inflammatory effects by promoting the formation of autoantibodies against both mitochondrial (Krysko et al., 2011; Wang et al., 2015) and nuclear (Barrat et al., 2005; Tsourouktsoglou et al., 2020) DNA, that can induce the activation of immune cells such as macrophage and neutrophils.

When considering also RETs and biofilms further issues arise: what conditions the exDNA released by root cap cells in the mucilage or while forming biofilms produces? Can they be either positive or negative for the host, either in roots or in microbial organisms, respectively? Could the co-secreted ECM have also the role of decreasing the bio-availability of extracellular self-DNA molecules to limit its potential inhibitory role, thus favoring the formation of RETs, or biofilms, or NETs, and forming self “exDNA traps,” thus limiting possible self-inhibitory effects? Although in the context of RETs and microbial biofilms a direct negative role of the exDNA in the matrices against the producing organisms has never been shown, the inhibitory effect of exDNA fragments has been demonstrated to be a general biological process in animal, plants, and bacteria (Mazzoleni et al., 2015b).

Therefore, a major issue remains to be considered on the possible roles of the ECM components in structuring and organizing exDNA. The trapping of exDNA in the ECM offers protective advantages against foreigner attacks, and, in parallel, limits its bio-availability in the environment as a free molecule, affecting its possible effects on the releasing organisms. In this framework the matrices could play an additional role in limiting the extracellular self-DNA self-inhibitory effects.

Studies addressing the roles of DNA in extracellular environments, and specifically in extracellular matrices formation, will shed further light on additional mechanisms and functionalities of these complex systems, ubiquitarian across different kingdoms.

## Author Contributions

EP, FM, and MC wrote the manuscript. MC conceived the effort and supervised the entire work. PT, PC, EA, and SM gave their intellectual contribution to the work. All the authors read and approved the work.

## Conflict of Interest

The authors declare that the research was conducted in the absence of any commercial or financial relationships that could be construed as a potential conflict of interest.
